# A New Strategy to Produce Hemp Fibers through a Waterglass-Based Ecofriendly Process

**DOI:** 10.3390/ma13081844

**Published:** 2020-04-14

**Authors:** Aurelio Bifulco, Brigida Silvestri, Jessica Passaro, Luca Boccarusso, Valentina Roviello, Francesco Branda, Massimo Durante

**Affiliations:** Department of Chemical Materials and Industrial Production Engineering (DICMaPI), University of Naples Federico II, P.leTecchio 80, 80125 Naples, Italy; aurelio.bifulco@unina.it (A.B.); jessica.passaro@unina.it (J.P.); luca.boccarusso@unina.it (L.B.); valentina.roviello@unina.it (V.R.); branda@unina.it (F.B.); mdurante@unina.it (M.D.)

**Keywords:** hemp fibers, waterglass, ecofriendly production, epoxy composites, silica coating, web-like structure

## Abstract

Natural fibers such as kenaf, hemp, flax, jute, and sisal have become the subject of much research as potential green or eco-friendly reinforcement composites, since they assure the reduction of weight, cost, and CO_2_ release with less reliance on oil sources. Herein, an inexpensive and eco-friendly waterglass treatment is proposed, allowing the production of silica-coated fibers that can be easily obtained in micro/nano fibrils through a low power mixer. The silica coating has been exploited to improve the chemical compatibility between fibers and the polymer matrix through the reaction of silanol groups with suitable coupling agents. In particular, silica-coated fibers easily functionalized with (3-Aminopropyl) triethoxysilane (APTS) were used as a filler in the manufacturing of epoxy-based composites. Morphological investigation of the composites through Scanning Electron Microscopy (SEM) demonstrated that the filler has a tendency to produce a web-like structure, formed by continuously interconnected fibrils and microfibrils, from which particularly effective mechanical properties may be obtained. Dynamic Mechanical Analysis (DMA) shows that the functionalized fibers, in a concentration of 5 wt%, strongly affect the glass transformation temperature (10 °C increase) and the storage modulus of the pristine resin. Taking into account the large number of organosilicon compounds (in particular the alkoxide ones) available on the market, the new process appears to pave the way for the cleaner and cheaper production of biocomposites with different polymeric matrices and well-tailored interfaces.

## 1. Introduction

Recently, natural fibers such as kenaf, hemp, flax, jute, and sisal have become the subject of much research as potential green or eco-friendly reinforcement composites, since they assure the reduction of weight, cost, and CO_2_ release with less reliance on oil sources [1,2,3]. The life cycle assessment (LCA) method supports the environmental, social and economic advantages through the use of such fibers [4].

Microfibrillated celluloses (MFC) were obtained for the first time via wood pulp through repeated passages in a high-pressure homogenizer [3,5,6,7,8,9]. Specifically, the fibers were disintegrated into fibrils and microfibrils through mechanical action. MFC, also known as nanofibrillated cellulose (NFC) and cellulose nanofibers or nanofibrils (CNF), were later produced in a diameter of 5 nm, from various natural sources [1,3,5,6] through homogenization, microfluidization, microgrinding and cryocrushing. There were, however, three severe drawbacks: mechanical energy consumption, fibers entanglement, and clogging of the mechanical apparatus [3,5,6]. A large amount of the produced CNF with some non-fibrillated residual fibers was obtained, with the degree of fibrillation dependent on the mechanical energy consumption [3,6].

Biological and chemical pre-treatments have made CNF production easier and more attractive for commercial applications. Mild cellulose hydrolysis, catalyzed by some enzymes, promotes fibrillation [3,5], whereas the introduction of negatively charged groups (through carboxylation via TEMPO-mediated oxidation or via periodate–chlorite oxidation, sulfonation and carboxymethylation) or positively charged groups (quaternization) on the cellulosic fibers [3,5] enhances delamination.

Alternative methods can be used to produce products of superior properties, which will hopefully be discovered [3,5].

Another severe drawback is the hydrophilic character of cellulose and the formation of a strong network held by hydrogen bonds [3,5,10,11,12]. For this reason, surface modification is necessary to obtain good dispersion and compatibility in non-polar polymer matrices. These methods are well described in the literature [3,5,10,11,12,13,14,15,16,17].

In this paper, a new pretreatment that allows hemp fibers with diameters ranging from tens of microns to tens of nanometers with the aid of a low power mixer is described. The method exploits recent findings [18] that demonstrated the formation of a silica-based coating, following the use of inexpensive and ecofriendly waterglass solutions. The silica-based coating demonstrated resistance to washing and was also able to act as a protective and thermal shield. Fourier Transform Infrared (FTIR) and solid-state Nuclear Magnetic Resonance (NMR) analysis strongly supported the formation of –C–O–Si– covalent bonds between the coating and the cellulosic substrate. In this paper it is shown that when effectively prolonging this eco-friendly process, the fabric becomes brittle and easily produces silica coated hemp fibers with the aid of a low power mixer. The silica coated fibers could be comfortably functionalized with (3-Aminopropyl) triethoxysilane (APTS) and then dispersed in epoxy resin. The functionalized fibers were easily dispersed in epoxy resin and, in a concentration of 5 wt%, strongly affected the glass transformation temperature (10 °C increase) and the storage modulus of the pristine resin.

Finally, it is worth remembering that nowadays, there is a large number of organosilicon compounds available on the market, allowing the easy compatibilization of silica and polymer matrices of very different chemical natures. Therefore, biocomposites with well-tailored interfaces and different polymeric matrices can be obtained through similar cheap, clean production, as described in the current paper, using an appropriate silane coupling agent (e.g., APTS).

## 2. Experimental

### 2.1. Materials

Sodium metasilicate (waterglass), 3-aminopropyltrimethoxysilane (APTS) hydrochloric acid and Ninydrin (37% ACS) reagents were purchased from Sigma-Aldrich (St. Louis, MO, USA). Plain weave hemp fabrics (grammage: 160 g m^−2^, supplied by MAEKO S.r.l., Milan, Italy) and a two-component epoxy resin system (SX10 by MATES S.r.l., Milan, Italy) were used for fabricating composite laminates. The two components are reported by the supplier to be modified bisphenol A resin and modified cycloaliphatic polyamines. The hemp fabric is reported by the producer to have been submitted to several treatments: 1) 1.5/2 h in 0.2% soap solution; 1.5/2 h in 0.2% soda Solvay solution; 1.5/2 h in 0.1% sodium hydroxide solution; 1.5/2 h in 1.5 gr/lt hydrogen peroxide solution.

The cellulose and lignin content were estimated to be equal to 70% and 5%–6% respectively.

### 2.2. Production of Functionalized Hemp Particles

Hemp fabric sheets were submitted to iterated soaking–drying cycles. In each cycle, the hemp fabric sheets were soaked for 20 min in a diluted (0.01 M) solution of waterglass (Na_2_SiO_3_) acidified up to Ph = 2.5 with hydrochloric acid. The sheets were left to dry for 15 min at 80 °C.

**Scheme 1:**
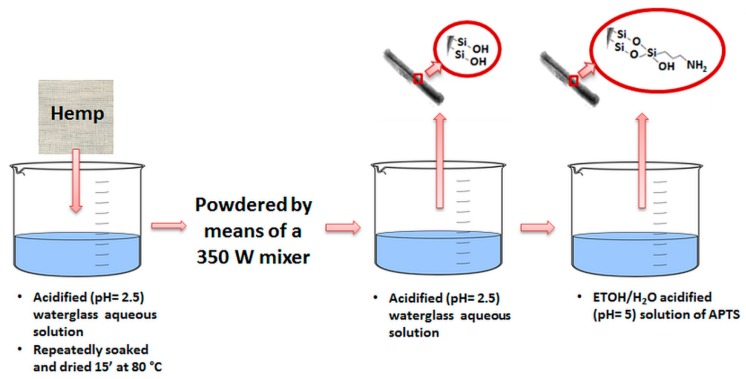
Overall scheme of the new fiber modification strategy to produce APTS functionalized filler.

After 30 cycles, the dry sheets could be easily torn and then reduced to powder with the aid of a low power (350 W) mixer (IMETEC S.p.A., Azzano San Paolo, Bergamo, Italy). When the motion was similar to wadding, water was added (i.e., 20 g of hemp in 100 ml of water) to successfully complete the grinding. The obtained pulp was dispersed in a waterglass solution 0.1 M acidified up to pH = 2.5 with hydrochloric acid and stirred for 24h (i.e., 20 g of hemp in 500 ml of solution). The suspension was left to settle and the supernatant was substituted with distilled water in a stirred condition. This operation was repeated three times. The hemp pulp was also washed three times in centrifuge with water/ethanol mixtures (50% in volume). After drying at 80 °C, the hemp was reduced to a powder in the 350 W mixer. The powders were dispersed in water and ultrasonicated (20 kHz, 900–1000 W, 30 min). The final product was washed (three times) with a solution of EtOH/Water (50/50, vol/vol) in centrifuge (10,000 rpm, 10 min, Thermo Fisher Scientific S.p.A., Waltham, MA, USA) and dried for 24 h at 40 °C. The functionalization with surface amino groups has been performed by dispersing the particles (indicated in the following as Hemp_SiO_2_) in a solution of EtOH/Water (80/20, vol/vol), containing 3-aminopropyltrimethoxysilane (APTS) (10 vol.%) and subsequently acidified up to pH = 5 with acetic acid. The functionalization occurs after only one soaking/drying cycle (soaking time/drying time = 20 min/10 min). The final product is then washed with a solution of EtOH/Water (50/50, vol/vol) through three centrifugation cycles (10,000 rpm, 10 min). Finally, the APTS functionalized fibers (indicated in the following as Hemp_SiO_2__APTS) were dried for 24h at 80°C. Scheme 1 shows a sketch of the new fiber modification strategy.

### 2.3. Synthesis of Composites

The synthesis of the composites epoxy/APTS functionalized hemp fibers was performed involving the following two steps:

1. Mixtures of epoxy DGEBA (Bisphenol A diglycidyl ether) and amino functionalized fibers, with weight percentages of hemp particles equal to 1, 2, 5 wt%, were stirred vigorously at 80 °C for 2 h in a closed system to allow the reaction between the primary amino groups and oxirane rings of epoxy resin.
2. The amount of hardener needed for the curing (26 wt% of the epoxy resin) was added to the mixture at room temperature and mixed for 5 min, subsequently poured into a Teflon® mold. The samples were cured for 24 h at 30 °C and post-cured for 4 h at 80 °C.

### 2.4. Scanning Electron Microscopy (SEM) with Energy Dispersive X-Ray Analysis (EDX)

A Leica Stereoscan 440 Microscope (20 kV) (Leica Microsystems Cambridge Ltd., Cambridge, UK), equipped with an energy Dispersive Analytical System (EDX) from Inca Energy 200, by using AZtecEnergy EDS Software (v2.1, Oxford Instruments, Abingdon, UK, 2006) was used.

### 2.5. Fourier Transform Infrared Spectroscopy (FTIR) 

The spectra were acquired with a Nikolet 5700 FTIR spectrometer (Thermo Fisher, Waltham, MA, USA) (KBr pellets) with a resolution of 4 cm^−1^ and 32 scans and Thermo Scientific™ OMNIC™ Software Suite (v7.2, Thermo Fisher, Waltham, MA, USA, 2005).

### 2.6. Ninhydrin Test

A small quantity of the APTS functionalized fibers is soaked into a solution of ninhydrin (2,2-dihydroxyindane-1,3-dione) EtOH/Water (80/20, vol/vol) solution. The presence of primary amino groups can be easily highlighted. In fact, it reacts with ninhydrin forming a Schiff base, with the appearance of a characteristic blue-violet-color [19].

### 2.7. Dynamic Mechanical Analysis (DMA)

DMA tests were carried out according to ASTM D4065-01 using a TA (RSA III) instrument in a three-point bending configuration, adopting an oscillation frequency of 1 Hz under controlled sinusoidal strain. The samples (50 × 12.5 × 3 mm^3^) were heated to 200 °C at 3 °C/min.

## 3. Results and Discussion

### 3.1. Preparation and Characterization of Hemp Fibers

Hemp fabrics were iteratively soaked into acidified waterglass solutions following the procedure described in Section 2.2 and sketched in Scheme 1. The treatment is similar to the one reported in a previous paper [18] where it was shown that the obtained silica-based coating was washing resistant and able to act as a protective and thermal shield. Fourier Transform Infrared (FTIR) and solid-state Nuclear Magnetic Resonance (NMR) analysis well supported [18] the formation of –C–O–Si– covalent bonds between the coating and the cellulosic substrate. The hemp fabric is also the same as the one used in the previous work [18] where SEM micrograph and Thermogravimetric Analysis (TGA) are reported. In the latest paper, the effect of reiterating the exposure is described and exploited to obtain micro/nano fibers.

**Figure 1:**
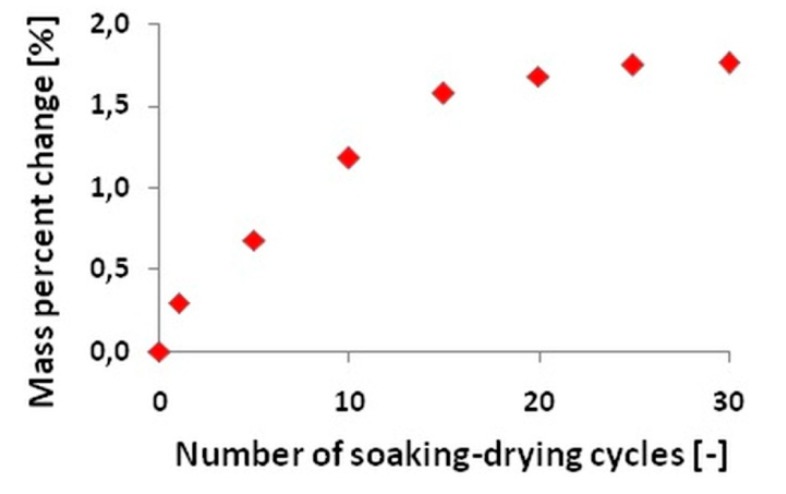
Percentage change as a function of the number of soaking–drying cycles.

Figure 1 shows how hemp fabric mass changes (as wt%) with the number of soaking–drying cycles. The treatment makes the fabrics progressively lose softness in terms of mechanical behavior.

After 30 cycles, the hemp fabrics became brittle enough to be easily torn up. Therefore, they may comfortably be reduced to powder with the aid of a low power (350 W) mixer, as previously described in the experimental section.

**Figure 2:**
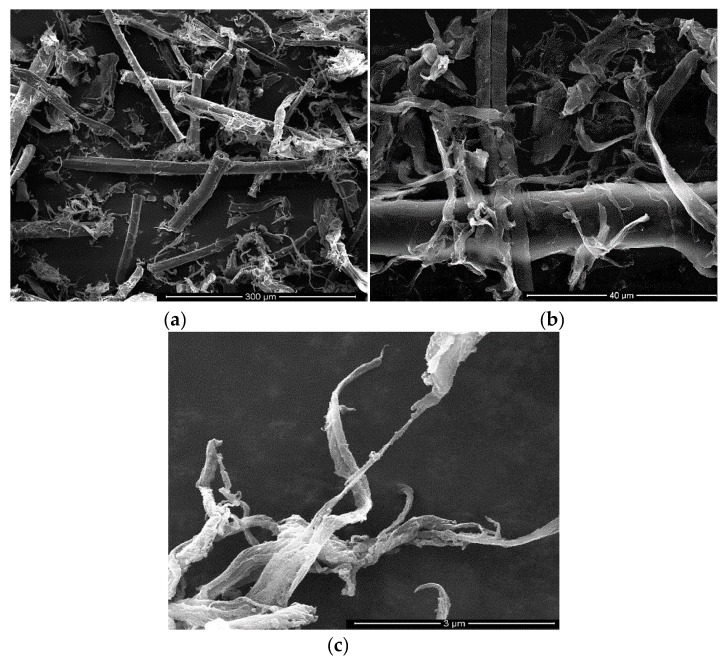
Micrographs of the fibers at different magnifications: (**a**) general overview at 300 µm (**b**) and increasing details at 40 µm (**c**) and 3 µm.

In Figure 2, the SEM micrograph (at different magnification) of the obtained powders is shown. As can be seen, fibers of diameter ranging from tens of microns to tens of nanometers are obtained. In order to explain the results, it is worth reminding that plant fibers do possess a hierarchical structure [20]. They consist of elementary fibers corresponding to single cells of 1–50 mm in length and 10–50 µm in diameter. The central lumen allowing water uptake is surrounded by several cell walls consisting of cellulose microfibrils (10–30 nm in diameter) embedded in a hemicellulose-lignin matrix. In particular, they differ due to the composition of the matrix and the orientation of the microfibrils. Each microfibril consists of 30–100 cellulose molecules in extended chain conformation.

Recently the authors [18] proved that the exposure of hemp fabrics to inexpensive and ecofriendly 0.1 M waterglass solutions allowed the formation of a well anchored silica-based coating, additionally resistant to washing. Fourier Transform Infrared (FTIR) Spectroscopy and solid state Nuclear Magnetic Resonance (NMR) analysis [18] demonstrated the formation of –C–O–Si– covalent bonds as a result of the condensation reaction on the silica coating/cellulose interface. Therefore, the above-described results may be due to the use of a lower concentration (i.e, 0.01 M) combined with an elongation of the exposure time (see Section 2.3) [18]. This methodology may allow a deep penetration of the waterglass aqueous solution through the hierarchical structure of hemp fibers. The formation of the silicate layer could be a result of the observed brittleness and effortless size reduction, in a low power mixer, to fibers of diameter from microns to tens of nanometers. The obtained silica coated hemp fibers were easily functionalized with APTS as described in Section 2.2.

**Figure 3:**
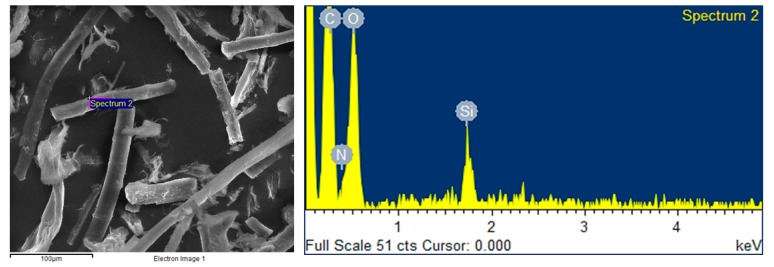
Micrograph of hemp fiber after APTS functionalization and EDX spectrum.

In Figure 3, the SEM micrograph of hemp fiber following APTS functionalization is shown. As can be seen, the general aspect of the fibers is similar to the one observed in Figure 2. Figure 3 shows the EDX spectrum confirming the presence of nitrogen on the surface of the fibers.

The presence of amino groups on the surface of the functionalized fibers is also well supported by the ninhydrin test and FTIR spectra. In fact, when a small quantity of the treated microfibers was added to the ninhydrin solution (see Section 2.6), the characteristic blue-violet color appeared.

**Figure 4:**
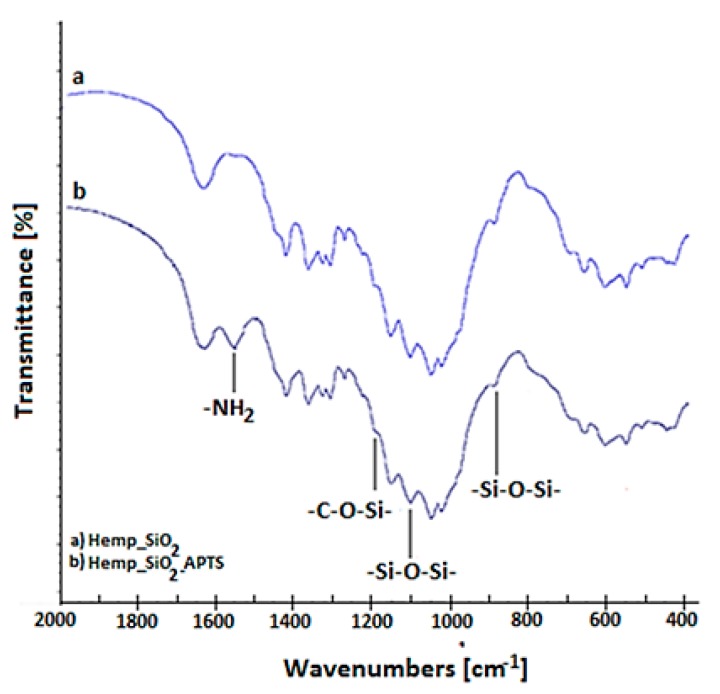
Spectra of (**a**) hemp fabric after functionalization with waterglass (Hemp_SiO_2_ sample), (**b**) the spectrum recorded after the functionalization with APTS (Hemp_SiO_2__ APTS sample).

Finally, the occurrence of the amino functionalization is further confirmed by FTIR spectra reported in Figure 4, which reports the spectra of hemp fibers (Hemp_SiO_2_ sample) before a) and after b) functionalization with APTS (Hemp_SiO_2__APTS sample). In detail, spectrum a) proves that the hemp fibers are produced with a silica coating. In fact, spectrum a) is similar to the one previously reported [18], thus differing from the hemp example for the presence of three bands; two of them are due to the well-known stretching vibration of silicate structure, the third one resulting from the condensation reaction between OH groups of hemp surfaces and silanols from silica resulting in the appearance of C-O-Si bonds [18,21,22]. As can be seen, the exposure to the APTS solution leads to the presence of the NH_2_ stretching vibration band on the FTIR spectrum [23].

### 3.2. Composite Epoxy/APTS functionalized Hemp Fibers

#### 3.2.1. SEM Observations

**Figure 5:**
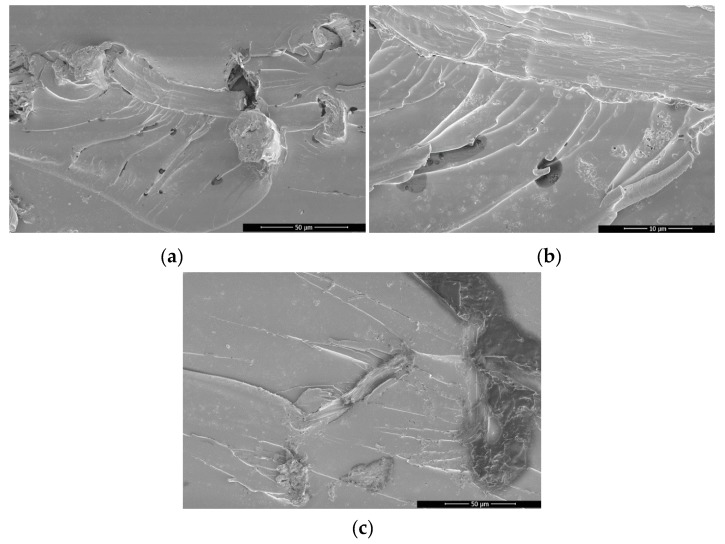
Micrographs of the composites epoxy/APTS functionalized hemp fibers: (**a**,**c**) 50 µm scale and its detail, (**b**) region at 10 µm.

Figure 5 shows SEM micrographs of the epoxy/APTS functionalized hemp fibers at different magnifications. Figure 5a suggests the formation of a web-like structure formed by fibrils and microfibrils. As better highlighted in Figure 5b, the epoxy phase appears to completely coat the entire surface of the fibers, due to a better chemical compatibility between the polymer matrix and the functionalized filler. The formation of this web-like structure could be ascribable to the good adhesion at the interphase performed by the chemical coupling between the amino groups of APTS and oxirane rings of the epoxy resin [3,24].

#### 3.2.2. DMA Results

**Figure 6:**
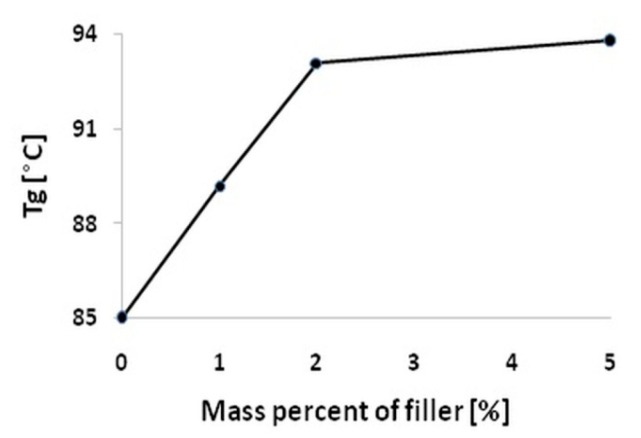
Glass transformation temperature of the composites epoxy/APTS functionalized hemp fibers as a function of composition.

**Figure 7:**
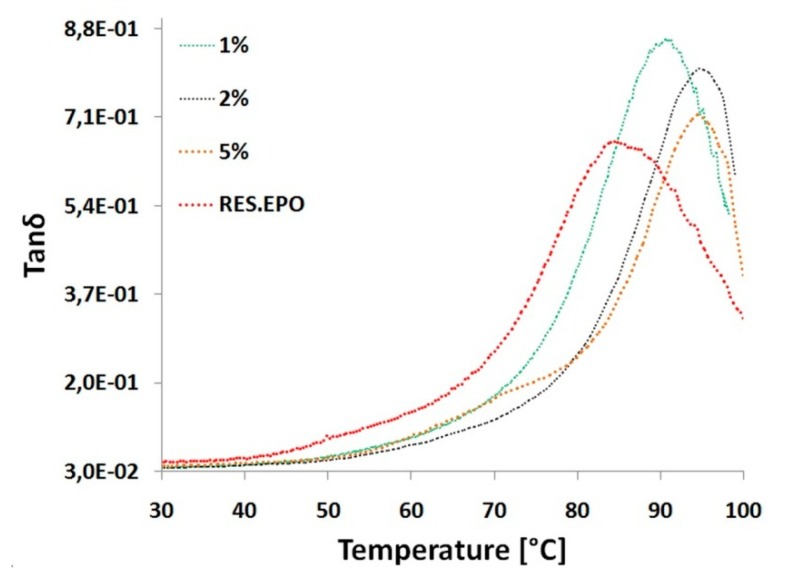
Comparison between the experimental values of Tanδ (calculated as storage and loss modulus ratio) for different volumetric percentage of filler content in the epoxy resin.

Figure 6 shows the plot of glass transformation temperature (Tg) as a function of filler composition. The Tg values were taken from DMA results as the Tanδ peak temperature (Figure 7). Thus, Tanδ values were calculated as the ratio between the loss (E’’) and storage (E’) modulus. It is possible to note that the composites do possess higher Tg than the neat epoxy resin, increasing with the hemp particle content. This is indicative of a good interaction at the fiber/epoxy interphase, in agreement with SEM micrographs (Figure 5).

**Figure 8:**
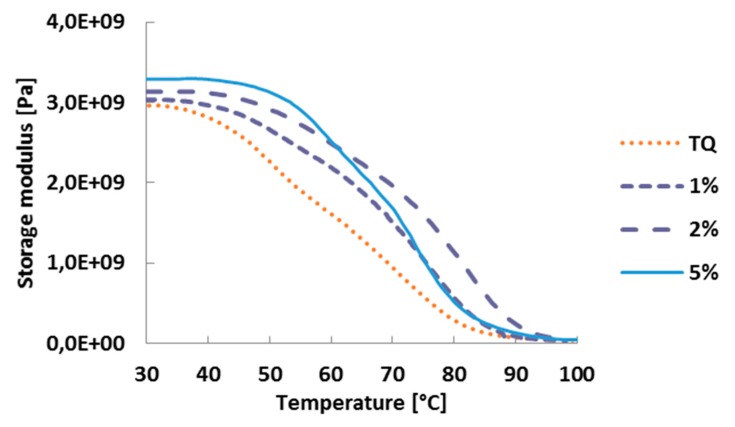
Storage moduli of the different specimens measured by DMA.

In Figure 8, the curves of the storage modulus (E’) in the function of temperature, obtained by DMA tests, are reported. It is possible to observe that the values of modulus begin to decrease after 50 °C.

Furthermore, in the tempurature range of 75–90 °C, the E’ curves result in an intense drop, which indicates a glass/rubbery transition.

**Figure 9:**
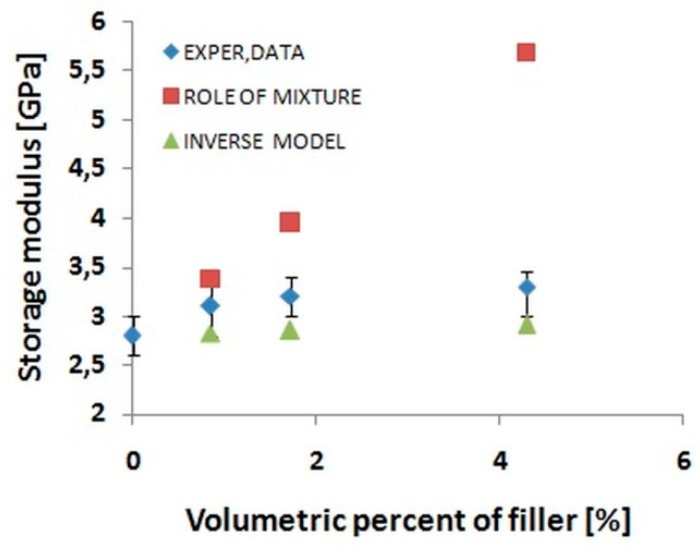
The difference between the experimental and theoretical values of moduli for different volumetric percentages of filler content in the epoxy resin.

Additionally, the trend of the average values of the storage moduli, reported in Figure 9, as a function of hemp content, clearly depicts that the incorporation of microfibers for the resin generates an increase in the modulus.

The experimental values were compared with the ones calculated through the “rule of mixtures” (valid for long unidirectional fibers) [25,26,27]:

(1) $$E=V_{f}E_{f}+V_{m}E_{m}$$

and the “inverse rule of mixtures” (valid in case of short fibers or fillers) [25,26,27]:

(2) $$\frac{1}{E}=\frac{V_{f}}{E_{f}}+\frac{1}{\frac{V_{m}}{E_{m}}}$$

where:

- E is composite modulus
- E_f_ is fiber or filler modulus
- E_m_ is the matrix modulus
- V_f_ and V_m_ are the volumetric fraction content of fibers and matrix respectively

In Figure 9, the average values of storage modulus at an environmental temperature are reported for the different values of volumetric content of fibers. In this diagram, a comparison with the results of the analytical formulae in the rule of mixture and inverse rule of mixture is reported.

The modulus increase expected by using Equation (2), i.e., “inverse rule of mixture”, would be negligible for the very low values of loading (1 and 2 vol%) considered in the present work.

From 1 wt% of microfibers loading, the modulus increases up to 10% compared to pristine polymer (see Figure 9). This may be ascribed to the establishment of a bond between macromolecules and microfibers, influencing the rigidity of the resin. Moreover, it is worth remembering that the “rule of mixture” is valid in the case of long unidirectional fiber-reinforced composites, while the “inverse rule of mixture” is valid for short fiber-reinforced composites. In particular, Figure 9 demonstrates that the two rules may may both be inadequate to fit the experimental storage modulus of composites containing a filler in a web-like structure, as already reported in literature [3].

## 4. Conclusions

When properly treated with a cheap and ecofriendly waterglass solution, hemp fabric effectively produces silica coated fibers of diameters ranging from tens of microns to tens of nanometers, with the aid of a low power mixer. The silica coated fibers can be functionalized with (3-Aminopropyl) triethoxysilane (APTS) and then dispersed in epoxy resin. SEM micrographs of the composites show a tendency to produce web-like structures formed by fibrils and microfibrils, which are continuously interconnected, from which particularly useful mechanical properties may be expected to result.

DMA analysis shows that the functionalized fibers, up to a concentration of 5 wt%, strongly affect the glass transformation temperature (10 °C increase) and the storage modulus of the pristine resin.

The reported results may be considered a case study. In fact, taking into account that many organosilicon compounds are available on the market, the silica coating of the hemp fibers is expected to be easily functionalized for the effective dispersion and tailoring of the interface in different polymer matrices. Thus, the proposed new process may be a cleaner alternative to the production of biocomposites with potentially improved mechanical performances.

## References

[1] Eichhorn S.J., Dufresne A., Aranguren M., Marcovich N.E., Capadona J.R., Rowan S.J., Weder C., Thielemans W., Roman M., Renneckar S. (2010). Review: Current international research into cellulose nanofibers and nanocomposites. J. Mater. Sci..

[2] Holbery J., Houston D. (2006). Natural-fiber-reinforced polymer composites in automotive applications. Jom.

[3] Siqueira G., Bras J., Dufresne A. (2010). Cellulosic biocomposites: A review of preparation; properties and applications. Polymers.

[4] Alves C., Silva A.J., Reis L.G., Freitas M., Rodrigues L.B., Alves D.E. (2010). Ecodesign of automotive components making use of natural jute fiber composites. J. Clean. Prod..

[5] Nechyporchuk O., Belgacem M.N., Bras J. (2016). Production of cellulose nanofibrils: A review of recent advances. Ind. Crop. Prod..

[6] Spence K.L., Venditti R.A., Rojas O.J., Habibi Y., Pawlak J.J. (2011). A comparative study of energy consumption and physical properties of microfibrillated cellulose produced by different processing methods. Cellulose.

[7] Turbak A.F., Snyder F.W., Sandberg K.R. (1983). Microfibrillated Cellulose, a New Cellulose Product: Properties, Uses and Commercial Potential.

[8] Turbak A.F., Snyder F.W., Sandberg K.R. (1983). Microfibrillated Cellulose. U.S. Patent.

[9] Herrick F.W., Casebier R.L., Hamilton J.K., Sandberg K.R. (1983). Microfibrillated cellulose: Morphology and accessibility. Proceedings of the Ninth Cellulose Conference.

[10] Hubbe M.A., Rojas O.J., Lucia L.A., Sain M. (2008). Cellulosic nanocomposites: A review. BioResources.

[11] Missoum K., Belgacem M.N., Bras J. (2013). Nanofibrillated cellulose surface modification: A review. Materials.

[12] Kalia S., Boufi S., Celli A., Kango S. (2014). Nanofibrillated cellulose: Surface modification and potential applications. Colloid Polym. Sci..

[13] Mann G.S., Singh L.P., Kumar P., Singh S. (2018). Green composites: A review of processing technologies and recent applications. J. Thermoplast. Compos. Mater..

[14] Gu H., Ma C., Gu J., Guo J., Yan X., Huang J., Zhang Q., Guo Z. (2016). An overview of multifunctional epoxy nanocomposites. J. Mater. Chem. C.

[15] Dharmalingam S., Meenakshisundaram O., Elumalai V., Boopathy R.S. (2020). An Investigation on the Interfacial Adhesion between Amine Functionalized Luffa Fiber and Epoxy Resin and Its Effect on Thermal and Mechanical Properties of Their Composites. J. Nat. Fibers.

[16] Joseph B.G., Rajan J.A., Jeevahan J., Mageshwaran G. (2019). Influence of alkaline treatment on improving mechanical properties of jute fiber-reinforced epoxy (LY556) composites. FME Trans..

[17] Sepe R., Bollino F., Boccarusso L., Caputo F. (2018). Influence of chemical treatments on mechanical properties of hemp fiber reinforced composites. Compos. B Eng..

[18] Branda F., Malucelli G., Durante M., Piccolo A., Mazzei P., Costantini A., Silvestri B., Pennetta M., Bifulco A. (2016). Silica treatments: A fireretardant strategy for hempfabric/epoxycomposites. Polymers.

[19] West R. (1965). Siegfried Ruhemann and the discovery of ninhydrin. J. Chem. Edu..

[20] Thomas S., Paul S.A., Pothan L.A., Deepa B. (2011). Natural fibres: Structure, properties and applications. Cellulose Fibers: Bio- and Nano-Polymer Composites.

[21] Silvestri B., Pezzella A., Luciani G., Costantini A., Tescione F., Branda F. (2012). Heparinconjugatedsilicananoparticlesynthesis. Mater. Sci. Eng. C..

[22] Passaro J., Russo P., Bifulco A., De Martino M.T., Granata V., Vitolo B., Iannace G., Vecchione A., Marulo F., Branda F. (2019). Water resistant self-extinguishing low frequency soundproofing polyvinylpyrrolidone basedelectrospun blankets. Polymers.

[23] Califano V., Sannino F., Costantini A., Avossa J., Cimino S., Aronne A. (2018). Wrinkledsilicananoparticles: Efficientmatrix for β-glucosidaseimmobilization. J. Phys. Chem. C.

[24] Nakagaito A.N., Yano H. (2005). Novel high-strength biocomposites based on microfibrillated cellulose having nano-order-unit web-like network structure. Appl. Phys. A-Mater..

[25] Mansor M.R., Sapuan S.M., Zainudin E.S., Nuraini A.A., Hambali A. (2013). Stiffness prediction of hybrid kenaf/glass fiber reinforced polypropylene composites using rule of mixtures (ROM) and rule of hybrid mixtures (RoHM). J. Polym. Mater..

[26] Del Borrello M., Mele M., Campana G., Secchi M. (2020). Manufacturing and characterization of hemp-reinforced epoxy composites. Polym. Compos..

[27] Haghi A.K., Zikov G.E., Zikov G.E., Bazylyak L.I., Aneli J.N. (2013). Applications of Polymers in Construction Technology: Effects of jute/polypropylene fiber on reinforcement soil. Polymers for Advanced Technologies: Processing, Characterization and Applications.

